# Parasitic Plants—Potential Vectors of Phytopathogens

**DOI:** 10.3390/pathogens13060484

**Published:** 2024-06-07

**Authors:** Stefan Savov, Bianka Marinova, Denitsa Teofanova, Martin Savov, Mariela Odjakova, Lyuben Zagorchev

**Affiliations:** Department of Biochemistry, Faculty of Biology, Sofia University “St. Kliment Ohridski”, 8 Dragan Tsankov blvd., 1164 Sofia, Bulgaria; stefan_savovidis@abv.bg (S.S.); bianca.marinova@gmail.com (B.M.); teofanova@biofac.uni-sofia.bg (D.T.); martin_savovidis@abv.bg (M.S.); modjakova@gmail.com (M.O.)

**Keywords:** haustoria, parasitic plants, plant virus, phytoplasma

## Abstract

Parasitic plants represent a peculiar group of semi- or fully heterotrophic plants, possessing the ability to extract water, minerals, and organic compounds from other plants. All parasitic plants, either root or stem, hemi- or holoparasitic, establish a vascular connection with their host plants through a highly specialized organ called haustoria. Apart from being the organ responsible for nutrient extraction, the haustorial connection is also a highway for various macromolecules, including DNA, proteins, and, apparently, phytopathogens. At least some parasitic plants are considered significant agricultural pests, contributing to enormous yield losses worldwide. Their negative effect is mainly direct, by the exhaustion of host plant fitness and decreasing growth and seed/fruit formation. However, they may pose an additional threat to agriculture by promoting the trans-species dispersion of various pathogens. The current review aims to summarize the available information and to raise awareness of this less-explored problem. We further explore the suitability of certain phytopathogens to serve as specific and efficient methods of control of parasitic plants, as well as methods for control of the phytopathogens.

## 1. Parasitism in Plants

### 1.1. Variety of Parasitic Plants

Parasitic flowering plants are a highly specialized group of vascular plants, which switch to a partially or fully heterotrophic lifestyle. Depending on the degree of loss of photosynthetic ability, they are commonly divided into hemiparasites (photosynthetic or partially photosynthetic) and holoparasites (non-photosynthetic), but the terms facultative and obligate are also used [1], and parasitic plants are also classified as obligate (cannot complete lifecycle without a host) and facultative. The other common classification is based on the site of vascular connection with the host plant, giving either root or stem parasitic plants. Of the nearly 5000 known species [2,3], the holoparasites account for about 10%, while the distribution between root and stem parasites is more even, 60 to 40%, respectively [1]. There are a total of 12 parasitic plant clades, corresponding to 12 independent evolutional events. However, over 90% of all parasitic plants fall into two nearly equal in species number (with over 2000 species) clades—the root hemi- and holoparasites of the family Orobanchaceae and the stem hemiparasites of the order Santalales [3]. The third in species number is the genus *Cuscuta*, family Convolvulaceae, with a little over 200 species. The other clades contain a few to several dozen species, often rare and highly specialized. Some exotic examples include the Rafflesiaceae, characterized by enormously large flowers, but a highly reduced vegetative part [4]. However, this list is far from exhaustive, and new species are described every year, mainly because root hemiparasites may remain unnoticed, or because some species are very difficult to distinguish morphologically [5].

Parasitic plants also differ significantly in their host preference. While some are highly specialized in a single, or several, host plant species, others are generalists and infect tens, or hundreds, of host plants from different families. Some notable examples of single-host specialists are *Orobanche cumana* Wallr., specifically infecting sunflower (*Helianthus annuus* L.) due to specific requirements of germination stimulants of the strigolactone group [6]. However, a switch in strigolactone responsiveness may expand the host range of *O. cumana* to other plant species [7]. The juniper dwarf mistletoe (*Arceuthobium oxycedri* (DC.) M. Bieb) is highly specific to several juniper species but is occasionally found on other tree species [8]. Therefore, it is highly unlikely that a parasitic plant is restricted to a single host species, but still, many are restricted to several closely related hosts. Well-known generalists are the members of the *Cuscuta* genus, as some were reported to have 200 (*Cuscuta europaea* L., *Cuscuta campestris* Yunck.) to nearly 350 (*Cuscuta epithymum* L.) host species in a single study [9]. At the individual level, Orobanchaceae and mistletoes tend to expand to a single or few host plant individuals; a single *Cuscuta* individual may simultaneously spread and infect multiple hosts. From a practical point of view, parasitic plants that are generalists and infect multiple hosts simultaneously would be more efficient pathogen vectors than host specialists, and such species that infect a single host (e.g., mistletoes).

Some parasitic plants are dangerous pathogens on their own. Probably the most devastating are the members of the *Striga* (witchweeds) genus (Orobanchaceae), namely *Striga hermonthica* (Delile) Benth., *Striga asiatica* (L.) Kuntze, and *Striga gesnerioides* (Willd.) Vatke. They cause nearly entire yield loss in various cultures, such as rice, sorghum, cowpea, maize, finger, and pearl millet in over 40 countries, mainly in Sub-Saharan Africa, causing over USD 10 billion in economic losses [10,11]. Broomrapes (*Orobanche* and *Phelypanche* genera) of the same family are probably second in terms of agricultural impact. At least nine species were reported as dangerous weeds in Europe, Asia, and North Africa [12]. They were reported to cause yield losses, anywhere from 10 to 100% in various legumes, carrots, tomatoes, etc. Out of over 200 *Cuscuta* species, relatively few are also considered important pests, with *C. campestris* being the top villain, causing significant losses in agriculture worldwide [13]. Some other parasitic plant species are also dangerous pathogens, but to a much lesser degree than the above-mentioned [13]. For the current overview, we focused mainly on representatives of Orobanchaceae and *Cuscuta* spp. (root and stem parasites, respectively), due to their economic impact.

The primary cause of the negative impact of parasitic plants on their hosts is the direct extraction of nutrients (both minerals and organic compounds) and water, thus decreasing the biomass and seed production of their hosts [14]. However, their impact may be much broader, including the modulation of below-ground communities [15], inhibition of host photosynthesis [16,17,18], etc. The most striking effect is the “bewitching” effect of *Striga* spp. on their hosts, consisting of wilting and chlorosis of the host in the very early stages of *Striga* infection [19]. This effect is not entirely understood, suggested to be caused by excessive amounts of exuded abscisic acid [20], and the disruption of other hormones [19], but is certainly far more devastating than the exhaustion of nutrients. Finally, parasitic plants may also represent a significant vector of other pathogens, such as viruses, phytoplasma, bacteria, and fungi, which they may acquire and transfer from one host to another, facilitated by the vascular connection.

### 1.2. Haustorium Properties

Regardless of their taxonomic position, host specificity, or evolutionary history, all parasitic plants require a vascular connection with their hosts, called the haustorium. Haustoria represent multicellular invasive organs, able to attach and penetrate host tissues, sometimes overcoming tissue incompatibilities, as many parasitic plants are able to parasitize non-related, taxonomically distant host species [21]. Haustoria of root parasites are divided into two types, lateral haustoria and terminal haustoria, which are specifically formed by the apical root meristem [22]. While lateral haustoria do not interfere with root tip elongation and allow the formation of multiple haustoria, the terminal haustoria lead to the termination of root growth [23]. Some plants can form both lateral and terminal haustoria, like it was shown in *Phelipanche ramosa* (L.) Pomel (=*Orobanche ramosa* L.) (Orobanchaceae), while others are capable of only lateral haustoria formation like *Phtheirospermum japonicum* (Thunb.) Kanitz (Orobanchaceae) [24]. To some extent, the presence of terminal haustoria is associated with obligate parasites, while lateral haustoria are present in hemiparasites and are regarded as evolutionarily older than terminal haustoria [25,26]. In the case of obligate parasites, like *Striga*, the terminal haustorium is formed first, immediately after germination, followed by the growth of adventitious roots and lateral haustoria [27]. In an older study [28], but still in use [27], terminal haustoria are also denoted as primary, while lateral haustoria are denoted as secondary. Haustoria of stem parasites are considered distinct from root haustoria and although similar in function, differ in molecular mechanisms of formation, like in *Cuscuta* spp. [26]. Other stem parasites may possess different haustoria in terms of structure and formation [1].

Roughly three distinct stages of haustorium formation can be defined—initiation (or prehaustorium), penetration into host tissues, and vascular connection establishment [21]. Before initiation, both root and stem parasites need to locate/recognize host tissue. In root parasites, this occurs through chemical signaling—the recognition of strigolactones, released in host root exudates to attract symbiotic mycorrhizal fungi [29,30,31]. Going further, obligate root parasites require strigolactone detection to germinate only in the presence of a suitable host [31], and are also definitive for the host specificity of certain root parasites [6,7]. Unlike them, members of the genus Cuscuta were not proven to require chemical stimuli for germination and employ light [32,33] and probably chemical [34] stimuli in host recognition. The initiation of the root haustorium (in Orobanchaceae) requires specific chemical compounds, released by the host, collectively called haustorium-inducing factors (HIFs), which were proved to be quite diverse [35,36]. Unlike them, haustorium initiation in *Cuscuta* further relies on specific light quality in combination with tactile stimuli [37]. The stage of penetration, regardless of the parasite taxonomy, involves a combination of hydrolytic and cell-wall-modifying enzymes [21,26,37]. Finally, the vascular connection is established, which is primarily xylem–xylem (xylem bridge), observed in all parasitic plants [21]. A linkage to the phloem of the host, however, seems to be less common and is observed in stem parasites *Cuscuta* spp., but also root parasites *Orobanche* spp., while not being presented in *Striga* spp. [38].

The main function of haustoria per se is the transfer of water, mineral nutrients, and photosynthates from the host to the parasite. It is often compared to grafting [39] and raises the problem of the complicated interaction between two genomes, connected via a non-interrupted connection [40]. In this context, the haustorium was reported on numerous occasions as a bidirectional transport highway for numerous different molecules, far more complicated than a simple source of nutrients for the parasite. In *Cuscuta* spp., this involves an open xylem–xylem connection, membrane-mediated phloem transport, and plasmodesmata connections between contacting parasite and host cell walls [41]. This allows extensive transport of macromolecules, including DNA [42], mRNAs [43,44], and proteins [45]. Similar results were reported and summarized for Orobanchaceae [46,47]. Concerning phytopathogens, there is an important question about the haustorial connection—whether it is a putative passage for such passengers, which it is [41,47], or if it could serve as a selective barrier, limiting their distribution. Although there is evidence that this connection may be selective at least for some chemical entities [48], overall, it is a putative passage for different phytopathogens.

## 2. Phytopathogens and Parasitic Plants

### 2.1. Major Phytopathogens

#### 2.1.1. Viruses

The group of plant viruses is a constantly growing group of non-cellular infectious agents, containing DNA or RNA and a protein coat, that cause multiple diseases in plants, with visual expression as leaf discoloration, stunted growth, mottling, and necrosis. The estimated economic effect of plant viral diseases reaches USD 30 billion per year in crop yield losses [49]. The list of known plant viruses exceeds thousands [50] and will further expand in the future, but not all are equally devastating and agriculturally important. Some efforts to classify plant viruses resulted in surveys, like the “Top 10 plant viruses in molecular plant pathology” [51] and “Top 10 economically important plant viruses” [52], based on their importance as agricultural pests and molecular study objects. While some are comparatively restricted in the host range, others are characterized by an extremely wide host range and wide range of vectors—for *Cucumber Mosaic Virus* (CMV), Bromoviridae, over 1200 host plant species from over 100 families, and 80 aphid species from 33 genera, were reported [51].

Therefore, it is not surprising that viruses were reported in parasitic plants. Historically, *Cuscuta* spp. was reported to be infected and transmit over 50 different viruses [53]. More recent reports are comparatively scarce, but confirm dodders as usual hosts for a variety of plant viruses. Of the six enlisted viruses (Table 1), one is a DNA virus of the Geminiviridae family (TYLCV) and all others are positive-strand RNA viruses from four different families. Reports on viruses in Orobanchaceae are even more scarce, although some of the viruses, found in *Cuscuta* [54,55], were also proved to be acquired by *Phelipanche aegyptiaca* (Pers.) Pomel (=*Orobanche aegyptiaca* Pers.) from host plants [56]. Overall, the recent literature lacks substantial studies of virus distribution among parasitic plants, also shown by the fact that some viruses were discovered accidentally during transcriptomics analyses [57].

#### 2.1.2. Phytoplasma

Phytoplasmas (*Candidatus Phytoplasma*: Mollicutes) are obligate intracellular bacteria that lack cell walls [63] and cause a variety of diseases [64]. They are phloem-mobile, e.g., they move within plants by the phloem traffic [65]. Common disease symptoms include little or yellow leaves, phyllody, witches’ brooms, etc., and could cause between 30% and 100% yield loss, depending on the crop plant [66]. Being phloem-mobile, it is not surprising that dodders are efficient reservoirs and vectors of several phytoplasma [67,68]. Phytoplasmas were also reported from several root parasites—*Ph. ramosa* as a host for tomato stolbur disease [69] and *Orobanche* spp. as a host for tomato big bud [70] and several *Orobanche*-specific phytoplasmas [71].

#### 2.1.3. Bacteria

Phytopathogenic bacteria are among the most devastating disease-causing pests, contributing to enormous yield losses in crop plants [72]. Unlike viruses and phytoplasma, they are not fully dependent on hosts to survive, and more often are soilborne pathogens, infecting plants through wounds, and moving through the xylem vessels. Several of the most destructible pathovars belong to the *Pseudomonas* and *Xanthomonas* genera, as well as notorious pathogens like *Ralstonia solanacearum* (Smith 1896) Yabuuchi et al., 1995, single-handedly contributing to over USD 1 billion annual losses, and *Erwinia amylovora* (Burrill 1882) Winslow et al., 1920 [73]. Important pathogens among Actinomycetes include members of the *Clavibacter* [74] and *Curtobacterium* [75]. Most of the studies involving phytopathogenic bacteria and parasitic plants are related to methods of control of the parasitic plants [76] and will be further discussed in detail hereinafter.

Interestingly, extracts from the members of the genus *Cuscuta* were shown to possess strong antibacterial activity against major phytopathogenic bacteria. For example, water extracts of *Cuscuta pedicellata* Ledeb. were proven in vitro as efficient control agents for fruit lesions, caused by *Xanthomonas campestris* (Pammel 1895) Dowson 1939 [77]. Similarly, organic solvent extracts of *Cuscuta reflexa* Roxb. were effective in the inhibition of *X. campestris* and several human pathogenic bacteria [78]. Ethanol extracts of several *Orobanche* species were also shown as efficient antibacterial agents against *Agrobacterium* and *Erwinia* [79]. *Cuscuta* spp. are particularly rich in bioactive compounds [80], as well as members of Orobanchaceae [81,82], so their in vitro antibacterial activity is not surprising, and additionally, they are regarded as highly tolerant to phytopathogenic bacteria [79].

#### 2.1.4. Fungi

The fourth major phytopathogenic group consists of fungi. According to some estimates, they account for nearly 80% of yield losses, caused by microbial pathogens [83]. Some of the most damaging species include *Magnaporthe oryzae* (T.T. Hebert) M.E. Barr, *Botrytis cinerea* Pers., and several *Fusarium* species, among others [84]. However, phytopathogenic fungi are a comparatively small portion of many fungi that live in symbiotic relations with plants, such as arbuscular mycorrhizal fungi, which are important and even critical for healthy plants [85], and endophytic fungi, which are also important players in combating other phytopathogens [86]. The relations between plants and fungi extend to the case of mycoheterotrophic plants, which are parasitizing fungi [87]. Both dodders [88,89] and Orobanchaceae [90,91,92] were shown to be rich in endophytic fungal biodiversity, and also several *Fusarium* spp. isolates showed promising results in terms of pathogenicity against the parasitic plants [93,94]. Phytopathogenic fungi were also the most exploited means of biological control of parasitic plants, which will be further discussed below.

### 2.2. Symptomatics

Since now, parasitic plants have been reported to be infected by multiple phytopathogens. However, they rarely exhibit visual symptoms of such infections, which is especially true for *Cuscuta* spp. and some holoparasitic members of Orobanchaceae, due to their simple, leafless morphology [1]. In terms of viral infections, they rarely experience visible phenotypic symptoms or are even reported as symptomless in various experiments [54,58,59]. Although some very early reports claim that *Cuscuta* spp. can suppress viruses from transmission to other plants [59], there is no recent confirmation of such inactivation and dodders can maintain high viral titer for years [59]. However, there are some reports that when parasitizing virus-infected hosts, some parasitic plants may exhibit a deformed phenotype, as in the case of *Ph. aegyptiaca*, infecting CMV-infected tobacco plants [95]. In terms of phytoplasma, Marcone [68] successfully employed *Cuscuta* spp. as vectors of several phytoplasmas, but did not report any visual symptoms in the dodder vector. Unlike them, *Orobanche* spp. showed clear evidence of phytoplasma infection, such as stem flattening and witches’ broom [71].

Fungal infection on *Cuscuta gronovii* Willd. was shown to cause observable symptoms, including the discoloration and shriveling of the stem, blighted portion, necrotic lesions, and tip necrosis [96]. *Cuscuta pentagona* Engelm. *Alternaria destruens* E.G. Simmons 1998 also causes necrotic spots and blights [97]. In *Orobanche* spp., different *Fusarium* isolates caused a variety of symptoms such as the inhibition of seed germination, wilting, and necrotic lesions on the stem and inflorescences [93,94]. Similar symptoms were also observed in *Striga* spp. as a result of *Fusarium* infection [98]. The seed germination of *Ph. aegyptiaca* was also found to be inhibited by *Pseudomonas* and *Bacillus* isolates [76]. Overall, symptomatics in parasitic plants seem to be much less pronounced and specific than in other plants, raising the necessity of molecular methods for the identification and screening of phytopathogens.

### 2.3. Detection Methods

General detection methods in plant pathology were recently summarized by Khakimov et al. [99] and could be roughly divided into traditional (e.g., visual inspection, microscopy, cultivation methods) and molecular (or modern, e.g., immunological, genetic, and mass-spectrometric). Visual observation highly relies on detectable symptoms, such as characteristic necrosis, chlorosis, etc., while microscopic methods may result in false identification, and also depend on the availability of certain pathogenic structures—for example, fungal spores or micellium [99]. Moreover, they are not always sufficient to provide the species identification of the pathogen and are somehow difficult to apply on parasitic plants, such as dodders and holoparasitic Orobancheacea, for the above-mentioned reasons. The most commonly employed modern methods for detection could be divided into immunological and molecular genetic methods. The immunological methods rely on the specific detection of an antigen of the pathogen by an antibody. Molecular methods employ PCR amplification and further sequencing of specific, taxon-discriminative DNA fragments. Phytopathogen detection in parasitic plants does not require any specific methods, differing from that established in the state-of-the-art. However, there are some peculiarities related to the scarce material of certain plant parts.

Plant virus detection is performed mainly by either commercialized ELISA kits [54] or quantitative RT-PCR with virus-specific primers, which also allow the determination of the viral titer [56,59]. Both approaches, however, allow the identification of particular viruses, selected in advance, which may underestimate the distribution of other viruses. Some more efficient methods, allowing the simultaneous detection of multiple, including unknown, viruses, as next generation sequencing (NGS)-based methods, loop-mediated isothermal amplification (LAMP), etc., were recently summarized by Mehetre et al. [100]. Being the most diverse group of phytopathogens, systematic viral detection would require a greater variety of methods. The detection of phytoplasmas commonly involves PCR amplification with phytoplasma-specific primers, of the DNA region, extending from the 5′ end of the 16S rRNA gene to the 5′ region of the 23S rRNA gene, and its amplicon may be either further analyzed by a Restriction Fragment Length Polymorphism (RFLP) analysis [68] or sequenced [101]. The typically low titers of phytoplasmas require the employment of nested PCR, or preferably quantitative real-time PCR, or LAMP.

The detection of bacterial and fungal pathogens is generally more straightforward than viruses and phytoplasmas. Most of them cause visible symptoms on parasitic plants (see above), are often visible by a common microscope, and could be cultivated on growth media in the absence of a host [102,103]. Moreover, the simultaneous NGS-based identification of thousands of bacterial and fungal taxa in a single sample, based on the sequencing of the variable regions of the 16S rRNA gene (for bacteria) and the nuclear ribosomal internal transcribed spacer (ITS) region (for fungi), is already a routine method [90].

## 3. Putative Routes of Transmission

### 3.1. Plant-to-Plant Transmission

**Figure 1:**
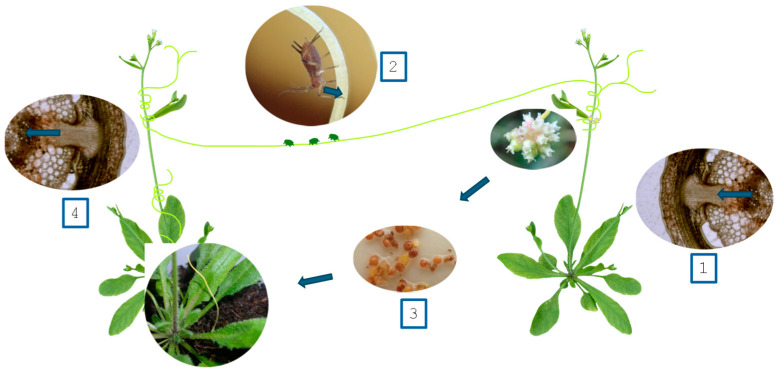
Putative transmission routes for phytopathogens, hypothesized for *Cuscuta* spp. as a vector. The parasitic plant acquires phytopathogens through the haustorial connection with an infected host plant (1) or from arthropods feeding on it (2). Subsequently, the phytopathogens are transmitted through the seed bank of *Cuscuta* (3) or directly (4) to a non-infected host plant.

As already discussed, the haustorial connection between parasitic plants and their hosts represents a hotspot of bi-directional macromolecular trafficking [46]. Along with interspecific plasmodesmata between the host and parasite phloem [56], it is also the major route by which parasitic plants could infect their hosts with different phytopathogens, especially viruses and phytoplasma [55,67,104]. This was not confirmed, but also might be predicted for bacteria, either actively or passively moving through plant vascular tissues [105]. The penetration nature of haustorium establishment is also important for phytopathogens, ensuring an efficient invasion route, and overcoming the plant cuticle. However, the main purpose of the haustorium is to extract molecules from the host, suggesting that parasitic plants may easily acquire phytopathogens from their hosts (Figure 1), and further distribute them to other hosts. Especially for dodders, their role in transferring a variety of signaling molecules between plants was already established [106]. More importantly, their generalism and simultaneous infection of multiple hosts from different species and families [9] also ensure the efficient spreading of pathogens between species.

However, the haustorial connection does not mean that all possible endophytes (incl. pathogens) would be efficiently distributed between the parasitic plant and the host. For example, the overlapping of endophytic fungi in *C. reflexa* and different hosts varied between 25% and 37% [88]. Parasitism of *C. campestris* affected the endophytic microbiome of *H. annuus*, but also with poor overlapping between the parasitic plant and the host plant [107]. Although different viruses can be acquired from the host, as in the case of *Ph. ramosa*, not all can successfully replicate in the parasite, and therefore not all can be further transmitted to other hosts [56]. There could be even specificity of virus acquisition and transmission among closely related parasitic plants—when a single virus (GLRaV-7) was detected in three different *Cuscuta* species, but only two were shown as vectors, and it was host-specific, e.g., one *Cuscuta* species may transfer the virus to one host, but not to another [59]. A similar specificity of phytoplasma transmission by *Cuscuta* spp. was shown by Marcone [68], where transmission rates differed greatly depending on both the particular phytoplasma and the dodder species, all of these suggesting that parasitic plants may be efficient, but very specific, phytopathogen vectors.

### 3.2. Arthropod-to-Plant Transmission

Hemipteran insects (Hemiptera: Linnaeus, 1758), topped by aphids, whiteflies, and leafhoppers, are considered the most important viral vectors, accounting for over 50% of the transmissions of known plant viruses [108]. Other arthropods such as mites, most notably eriophyid mites (Eriophyidae: Nalepa, 1898), are also known vectors of viral diseases [109,110] and nematodes are also significant vectors [111]. Similarly, hemipterans are also the most important vectors of phytoplasma [112,113]. Unlike these two groups, phytopathogenic bacteria and fungi are less commonly but not unlikely to be transmitted by arthropods [114,115,116].

As much as any other plant, parasitic plants are also associated with multiple arthropods that either feed or parasitize on them (Table 2). As clearly shown, both dodders and Orobanchaceae are subjected to feeding and parasitism by different groups of arthropods, potential vectors of phytopathogens. Moreover, it was also proven that molecules, such as mRNAs, could be transferred from the arthropods to the host plant [117]. Although it seems that the arthropod–parasitic plant–host plant route of phytopathogen transfer is not significant, as the insects may directly feed and infect the host plants, it must be noted that some arthropods are more or less specific to parasitic plants [118,119,120], or might not feed equally on the multiple hosts of the parasitic plant, or feed preferentially on the parasitic plant [121] as in the case of *Metcalfa pruinose* (Say, 1830), which fed exclusively on *C. campestris* when presented, but also attacked the host plant in the absence of the parasite. This will at least improve the chance of a phytopathogen to infect multiple plant species.

Although there is no experimental evidence of parasitic plants, serving as mediators of phytopathogens from arthropods to other plants, this potential route of transmission (Figure 1) seems plausible and requires more studies to assess the impact of both dodders and root parasites.

### 3.3. Seed Transmission

Most seeds of parasitic plants are characterized by long persistence in soil, where they can stay dormant for decades, waiting for the proper host to appear. This is mostly true for Orobanchaceae [132], which in combination with a large number of seeds, produced by a single plant, up to 200,000 in *Ph. ramosa* [133], gives a potentially enormous, long-term reservoir for various pathogens. *Cuscuta* spp. are also prominent seed producers, and although they do not require specific chemical compounds for germination, they are characterized by soil longevity and continuous germination over decades [134]. Such an enormous and persisting seed bank also represents a potential reservoir of numerous seed-borne pathogens from virtually every group [135], including fungi, bacteria, and viruses. Phytoplasmas are also frequently found in seeds of infected parents; the seed transmission of phytoplasmas is considered unlikely [112].

Besides some scarce reports [54], some of which are quite old [136], the potential of parasitic plant seeds to serve as a reservoir of plant viruses, transmitted to generations, is highly unexplored. The bacterial and fungal microbiota of root parasitic plants are much better studied and were shown to contain opportunistic, or obligate, phytopathogens as in *Ph. ramosa* [103,137] and *Cistanche phelypaea* (L.) Cout. [138]. Such pathogens are mostly soilborne and host plants are in contact with them anyway. However, the role of parasitic plants may be in the facilitation of pathogen penetration, simultaneously with the haustorium formation.

## 4. Phytopathogens as Biocontrol Agents of Parasitic Plants

Control of economically important parasitic plants is among the important aspects of contemporary agricultural practice, often complicated by their similarities to their hosts [12,13]. Being plants of their own, there is also a possibility that chemical control agents will affect their hosts equally, or even to a greater extent than the target parasites [139]. To overcome these limitations, several phytopathogens were studied and successfully applied as biocontrol agents, assuming they are highly specific to the parasite and affect neither host crop plants nor other plant species in proximity.

Both bacteria and fungi were extensively studied as potential biocontrol agents, and mostly isolated from natural sources, e.g., parasitic plants with visual symptoms of a disease. In *Cuscuta* spp., several species were found to be effective. One promising and already patented bioherbicide, specifically for dodder control, is *A. destruens* Strain 059 [97,140]. It was shown to affect a variety of dodder species, but it is not infecting other plant species, thus being a promising biocontrol agent. Some other promising results were shown for *Fusarium incarnatum* (Desm.) Sacc., *Alternaria dianthicola* Neerg., and *Curvularia pallescens* Boedijn. isolates from *C. gronovii* [96]. Of the bacterial pathogens, several *Bacillus* species were also shown to inhibit *Cuscuta* seed germination [141].

The inhibitory effect of bacterial isolates on Orobanchaceae members seemed to be much more extensive. These include the inhibition of radical elongation in *Ph. aegyptiaca* and *Orobanche cernua* Loefl. by two *Pseudomonas* and two *Bacillus* species [142], and in *Orobanche crenata* Forssk. and *Orobanche foetida* Poir. by two *Pseudomonas* species [143]. Besides their inhibitory activity on the parasitic plants, they also showed a positive effect on the growth of the host plants. The isolation of particular pathogenic strains, however, involves laborious screening of hundreds of strains. However, the most promising results for control of root parasitic plants were acquired with fungal pathogens of the *Alternaria* and *Fusarium* genera in both *Orobanche* spp. and *Striga* spp. The most exploited is probably *Fusarium oxysporum*, whose highly specific strains are efficient selective control agents for both the germination and growth of *Striga* [144,145,146,147], but also *Orobanche* spp. [148]. Other *Fusarium* species were also shown to be effective and selective pathogens on *Orobanche* [93,102]. There are hundreds of reports on the isolation and application of such fungal isolates as biocontrol agents on root parasitic plants, underlining the importance of such pathogens for food production security, especially in the poorest regions of Africa. One major advantage of such a bioherbicide is the possibility to propagate without any special equipment, so it is readily available to farmers in distant and poor regions [147]. However, most wild strains of putative phytopathogens are insufficiently virulent to be effective bioherbicides. The inhibitory effect of phytopathogens may be exerted by the direct infection of the plants, but also by the secretion of exometabolites, which specifically inhibit seed germination. Such exometabolites may be specific compounds [149], but also common metabolites such as amino acids, produced in excess [150]. The selection of an efficient hypervirulent biocontrol agent may be enhanced by the identification of such exometabolites and screening of multiple strains for the excessive secretion of such metabolites.

## 5. Conclusions and Future Perspectives

Although there is certain evidence that parasitic plants may serve as efficient vectors of phytopathogenic viruses, phytoplasmas, bacteria, and fungi, this aspect of their biology is highly understudied. This is because they often lack specific symptoms, but also because most of the efforts in studying parasitic plant biology were devoted to mechanisms of parasitism and methods of control, rather than phytopathogens. The available information is fragmented and does not allow a complete picture of the transmission routes, molecular mechanisms, and overall distribution of phytopathogens among the most economically damaging parasites of the *Cuscuta* genus and Orobanchaceae family. Besides the additional harm that transmitted phytopathogens add to the negative effect of parasitic plants on crop plants, they also represent a promising means of biological control, and more research on the overall endophytic diversity in both root and stem parasites is needed, to identify potential candidates for bioherbicides. Contemporary molecular methods, such as NGS-based DNA barcoding, offer the necessary tools for the high-throughput characterization of microbial diversity, associated with parasitic plants.

## Figures and Tables

**Table 1:** Recent examples of plant viruses, detected in parasitic plants.

Species	Parasitic Plant	Reference
TYLCV, CMV	*Cuscuta campestris*	[54]
TRV	*Cuscuta* spp.	[58]
GLRaV-7	*Cuscuta* spp.	[59]
LChV-1	*Cuscuta europaea*	[60]
PVY	*Cuscuta reflexa*	[55]
SaPlV1/2	*Striga hermonthica*	[57]
CMV, ToMV, PVY, TYLCV	*Phelipanche aegyptiaca*	[56]
PSTVd	*Orobanche ramosa*	[61,62]

Abbreviations: TYLCV—Tomato Yellow Leaf Curl Virus; CMV—Cucumber Mosaic Virus; TRV—Tobacco Rattle Virus; GLRaV-7—Grapevine Leafroll-Associated Virus-7; LChV-1—Little Cherry Virus-1; PVY—Potato Virus Y; SaPlV—Striga-Associated Poty-Like Virus; ToMV—Tomato Mosaic Virus; PSTVd—Potato Spindle Tuber Viroid.

**Table 2:** Non-exhaustive list of arthropods, associated with different parasitic plants.

Group	Species	Parasitic Plant	Reference
Hemiptera: Aphididae Hemiptera: Flatidae Hemiptera: Lygaeidae Diptera: Agromyzidae Coleoptera: Curculionidae	*Aphis fabae* *Myzus persicae* *Smynthurodes betae* *Geoica utricularia* *Metcalfa pruinosa* *Oxycarenus hyalinipennis* *Melanagromyza cuscutae* *Phytomyza orobanchia* *Smicronyx* spp.	*Cuscuta lupuliformis* *Cuscuta campestris* *Cuscuta australis* *Phelipanche ramosa* *Orobanche foetida* *Cuscuta campestris* *Cuscuta campestris* *Cuscuta* spp. *Orobanche* spp. *Cuscuta* spp. *Striga* spp.	[122] [123] [117,124] [125] [126] [121] [123] [118,127] [119,128,129] [120,130] [131]

## Data Availability

Not applicable.
